# Candidate protein markers for radiation biodosimetry in the hematopoietically humanized mouse model

**DOI:** 10.1038/s41598-018-31740-8

**Published:** 2018-09-10

**Authors:** Younghyun Lee, Monica Pujol Canadell, Igor Shuryak, Jay R. Perrier, Maria Taveras, Purvi Patel, Antonius Koller, Lubomir B. Smilenov, David J. Brenner, Emily I. Chen, Helen C. Turner

**Affiliations:** 1 Center for Radiological Research, Columbia University Medical Center, New York, NY 10032 USA; 2 Herbert Irving Comprehensive Cancer Center, Proteomics Shared Resource, Columbia University Medical Center, New York, NY 10032 USA; 3 Department of Pharmacology, Columbia University Medical Center, New York, NY 10032 USA

## Abstract

After a radiological incident, there is an urgent need for fast and reliable bioassays to identify radiation-exposed individuals within the first week post exposure. This study aimed to identify candidate radiation-responsive protein biomarkers in human lymphocytes *in vivo* using humanized NOD scid gamma (Hu-NSG) mouse model. Three days after X-irradiation (0–2 Gy, 88 cGy/min), human CD45+ lymphocytes were collected from the Hu-NSG mouse spleen and quantitative changes in the proteome of the human lymphocytes were analysed by mass spectrometry. Forty-six proteins were differentially expressed in response to radiation exposure. FDXR, BAX, DDB2 and ACTN1 proteins were shown to have dose-dependent response with a fold change greater than 2. When these proteins were used to estimate radiation dose by linear regression, the combination of FDXR, ACTN1 and DDB2 showed the lowest mean absolute errors (≤0.13 Gy) and highest coefficients of determination (R^2^ = 0.96). Biomarker validation studies were performed in human lymphocytes 3 days after irradiation *in vivo* and *in vitro*. In conclusion, this is the first study to identify radiation-induced human protein signatures *in vivo* using the humanized mouse model and develop a protein panel which could be used for the rapid assessment of absorbed dose 3 days after radiation exposure.

## Introduction

In the event of a radiological attack or accidental exposure, it will be necessary to quickly identify exposed victims from non-exposed groups and predict their received dose for effective medical treatment. In a mass-casualty scenario, a large number of people can be exposed to a wide range of radiation doses. It will be crucial to collect and analyze human biofluids (such as blood, urine, saliva) as soon as possible within the first week for accurate dose prediction and early triage decision. The applicability of potentially available biodosimetry methods for triage in large-scale radiation events was recently assessed^1^. Based on an updated comparative framework of six biodosimetry methods, increased population size, along with severely compromised infrastructure will increase the time needed to perform triage, which in turn will decrease the usefulness of many time intensive dosimetry methods. Therefore, this highlights the challenging need for the identification and development of potential diagnostic biomarkers for use as radiation biodosimeters of human exposure *in vivo* to ionizing radiation in terms of time- and dose-dependent response and high throughput capability, days after initial radiation exposure^2,3^. The rapid immunodetection of radiation responsive protein markers have potential as a useful diagnostic tool for the mass-screening of potentially exposed individuals. Earlier studies have suggested various radiation responsive proteins as candidate biomarkers for radiation biodosimetry^4–7^. However, the fact that DNA damage related proteins such as ATM and H2AX show increased amounts or changes in phosphorylation states within 24 h after exposure, limits their use as radiation biodosimeters for extended time points after initial radiation exposure^4^. Recently, Hall and colleagues^5^, provided a roadmap for developing biomarkers of radiation exposure from discovery to implementation to summarize the current status of proposed ionizing radiation biomarkers for epidemiological studies. This extensive review highlighted that most potential biomarkers remain at the discovery stage and require robust validation studies.

To date, the development and validation of radiation biomarkers *in vivo* has relied heavily on mouse models and more recently non-human primate (NHP) models due to the limitation in obtaining appropriate human samples^8,9^. Few studies have systematically validated radio-responsive human protein markers *in vivo* for dose- and time-response after radiation exposure. Radiation responsive plasma proteins such as Flt3 ligand (Flt3L), serum amyloid A (SAA) and Interleukin-6 (IL-6) have been studied in mouse models as markers of acute radiation syndrome and radiation exposure up to a week^10,11^. Using the NHP model, C-reactive protein (CRP), SAA, IL-6, Flt3L protein biomarkers expressed in NHP plasma have been proposed as early indicators of dose assessment and radiation-induced injury up to ~7 days post irradiation^12,13^. Recently, proteomics-based technology has been used to discover novel biomarkers for radiation biodosimetry in NHP models^14,15^.

The humanized mouse model provides an alternative model to study *in vivo* human biological response. This model is being increasingly used as a preclinical model in multiple biological fields including infectious diseases, immunology, cancer, regenerative medicine, hematology, and autoimmunity^16–18^. NOD*-scid IL2rγ*^*null*^ mice (NOG/NSG) are known to support higher engraftment with human CD34+ hematopoietic stem cells (HSCs) compared to BALB/c and CB17 scid strains^16^. The transplantation of HSCs derived from bone marrow, umbilical cord blood (UBC), or G-CSF–mobilized peripheral blood leads to the development of human hematopoietic progenitor and differentiated cells in the mouse bone marrow, spleen and thymus, culminating in a functional human immune system^17,19–21^. Recently, Wang *et al*. investigated the effects of ionizing radiation on human HSC cells^22,23^. Their results showed that radiation exposure induces DNA damage observed as γ-H2AX foci formation in the HSCs and promotes their aging-like phenotypic changes in the bone marrow microenvironment.

**Figure 1:**
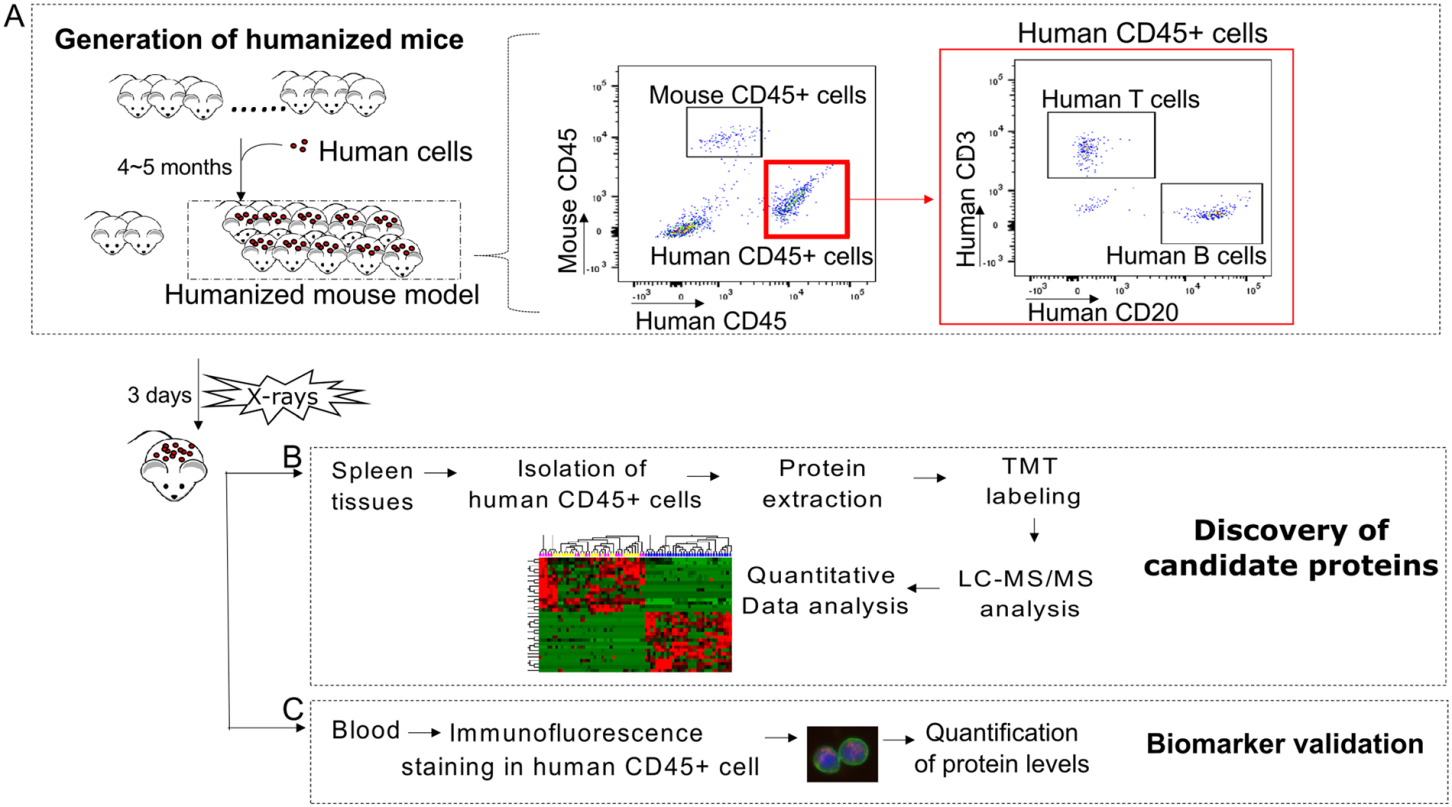
Experimental workflow to identify and validate candidate protein markers for radiation exposure using humanized mouse model. (**A**) Humanized mice were generated by injecting commercially available human cord blood CD34+ cells through the tail veins of NSG mice. Human cell engraftment was determined by measuring human CD45, human CD20, and human CD3 positive cells by flow cytometry 4–5 months after transplantation. Representative flow cytometry plots show the engraftment of human cells in non-irradiated humanized mice and hematopoietic reconstitution with human T and B cells in humanized mice. Humanized mice were then whole body-irradiated with X rays and the mouse spleen and blood were collected 3 days after irradiation. (**B**) Human cells were isolated from spleen tissues and analysed with isobaric tandem mass tag (TMT) labeled LC-MS/MS global proteome profiling. (**C**) Candidate protein expression levels were quantified in human CD45+ lymphocytes by immunofluorescence staining.

In the present study, we used the Hu-NSG mouse model to identify novel radiation-responsive candidate protein biomarkers in human lymphocytes *in vivo* for biodosimetry developmental studies. We applied shotgun proteomics to evaluate proteome-wide changes in human CD45+ B and T cells, 3 days after X-ray exposure (Fig. 1; workflow). Presented here are our top 4 protein candidate biomarkers and a panel of radiation responsive proteins to predict absorbed dose.

## Results

### Reconstitution of human hematopoietic cells in humanized mice

Ninety-seven percentage of recipient NSG mice showed successful engraftment 4–5 months after injection of HSCs. Figure 1A shows representative flow cytometry plots for human hematopoietic cell reconstitution from a humanized mouse tail blood prior to irradiation. The humanized mice (n = 33) generated in this study had 61.3 ± 17.9% human CD45+ cells including human B and T cells. Blood cell characteristics for each individual humanized mouse are detailed in Supplementary Table S1. Supplementary Fig. S1 shows the total number of human CD45+ cells across the three dose groups before and after irradiation. The results show similar proportion of human B and T cells with no statistically significant difference (*p* > 0.05) across each group before irradiation (Supplementary Fig. S1A). Since X rays dramatically decreased human lymphocyte cell number (Supplementary Fig. S1B), flow-sorted human CD45+ cells from mice injected with the same human HSCs were pooled in order to obtain sufficient amounts of protein for proteomic analysis.

### Protein signatures in X-irradiated human lymphocytes 3 days after exposure

**Figure 2:**
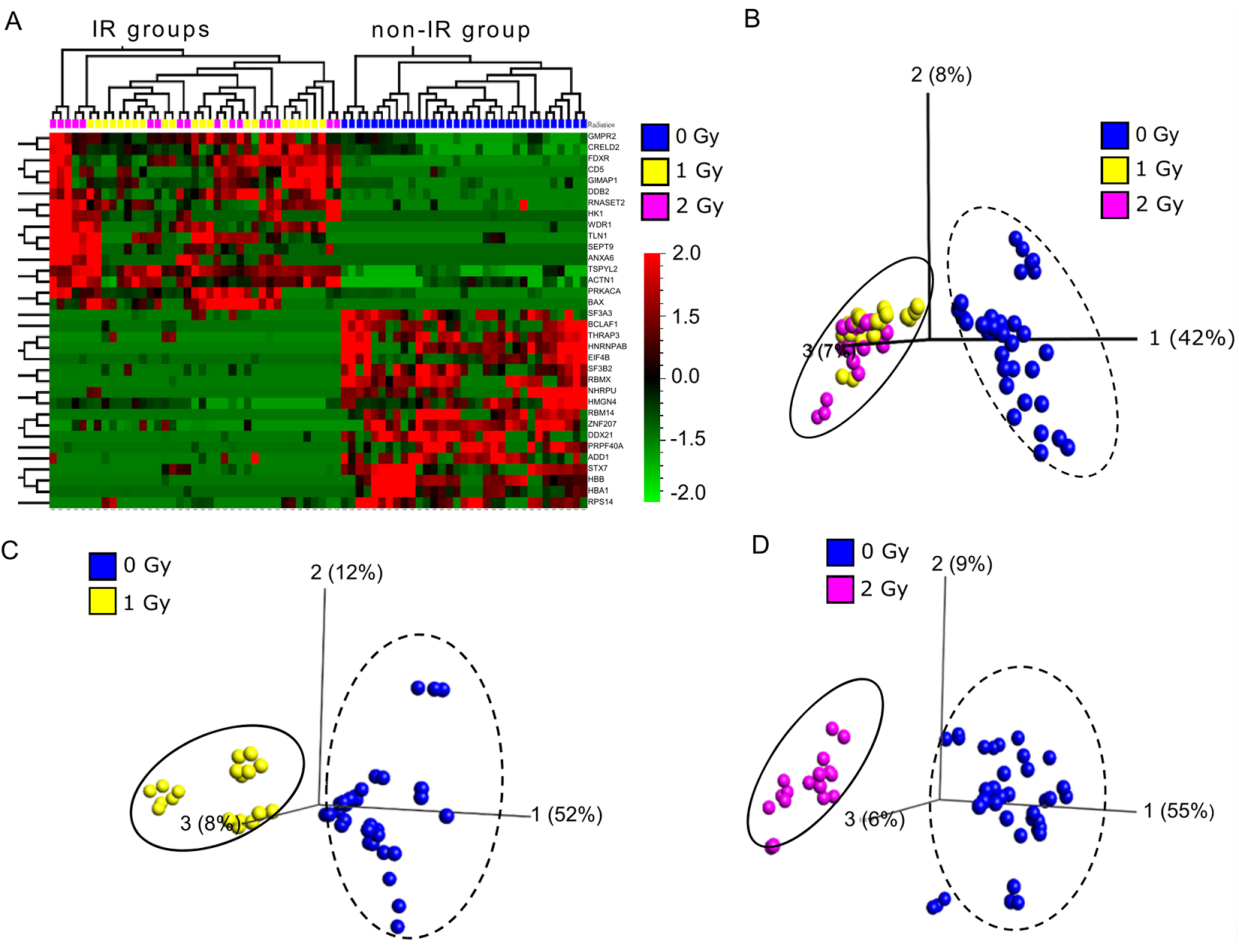
Statistical analysis of the proteome from human lymphocytes, isolated from humanized mice 3 days after irradiation. (**A**) Heat map showed a distinct protein signature between the non-irradiated group and irradiated groups at 0.001% of the false discovery rate (FDR) using ANOVA. Principal component analysis (PCA) plots showed comparisons of (**B**) three different dose groups (0, 1 and 2 Gy) at 0.001% FDR, (**C**) two dose groups (0 Gy and 1 Gy) at 0.010% FDR and, (**D**) two dose groups (0 Gy and 2 Gy) at 0.023%. Black dashed circle and solid circle represent clusters of non-irradiated and irradiated groups, respectively. A detailed protein list is included in Supplementary Table S2.

In total, 3,376 human proteins were identified based on 5% false discovery rate (FDR) at the protein level. In comparison of three different radiation dose groups, 34 proteins were found to be statistically significant at 0.001% FDR (Supplementary Table S2). Hierarchical clustering analysis and Principal Component Analysis (PCA) of these 34 proteins showed that the protein expression profiling can be separated into non-irradiated and irradiated clusters (Fig. 2A,B). Comparisons between 0 Gy vs 1 Gy (0.010% FDR; Fig. 2C) and 0 Gy vs 2 Gy (0.023% FDR, Fig. 2D) revealed a good separation according to radiation exposure and identified further 12 radiation responsive proteins.

### Selection of candidate protein markers for radiation biodosimetry

**Figure 3:**
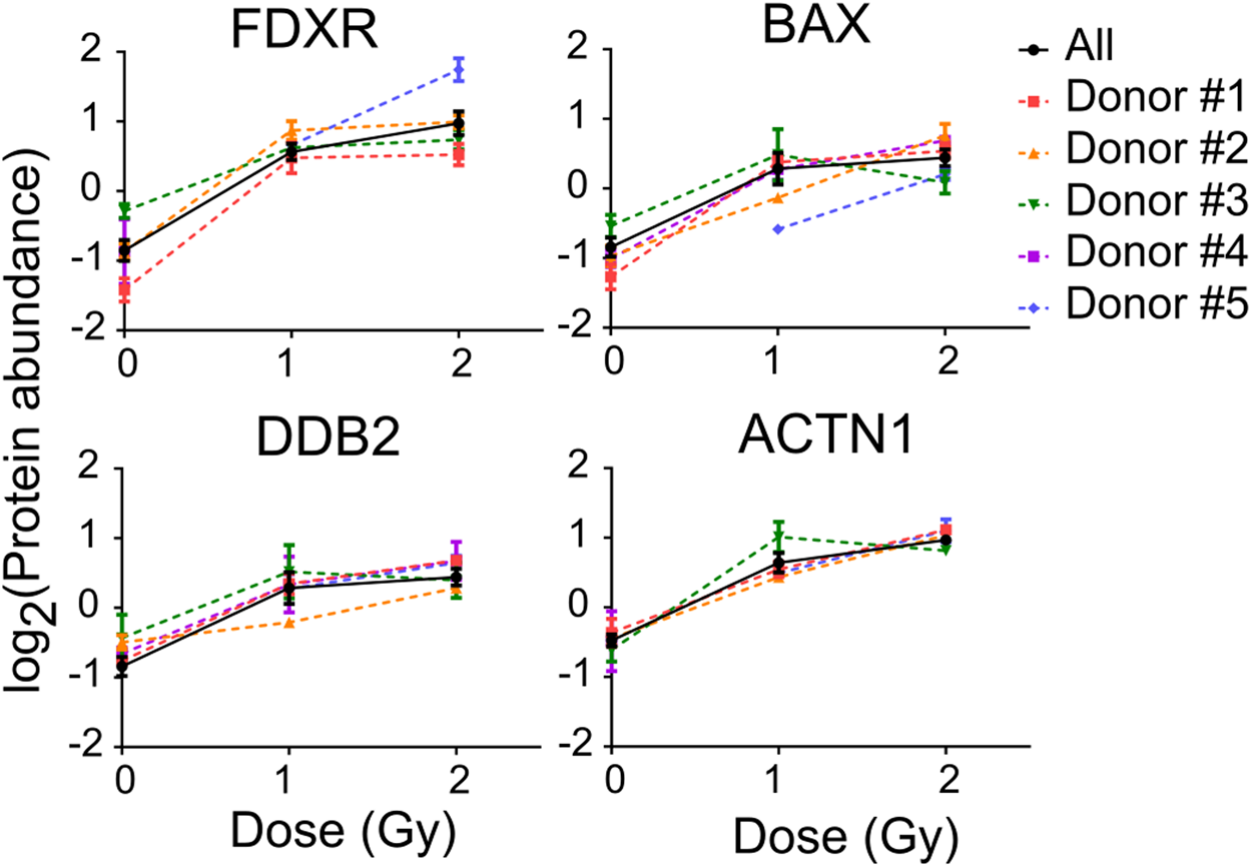
Radiation-induced changes in candidate protein expression in human lymphocytes isolated from humanized mice. Human lymphocytes in humanized mice were derived using stem cells from 5 different human donors, and isolated from humanized mice spleen 3 days after irradiation. After pooling samples to obtain sufficient cells, a total of 21 samples (0 Gy, n = 7; 1 Gy, n = 7; 2 Gy, n = 7) were analyzed for proteomic analysis. Dose response relationship of the best candidates (FDXR, BAX, DDB2, and ACTN1) in cells from each human donor is illustrated. Protein abundance quantified using TMT labeling was plotted by irradiation dose. Individual dose response curves from five human donors were plotted in dashed lines and colored symbols. Error bars in dose response curve of each donor were calculated from replicate measurements of pooled mice. Solid black line and circles depict the average dose response relationship. The error bar in averaged curve was calculated from data from 5 different donors. Data indicate mean ± SEM.

The 46 differential radiation responsive proteins identified above are presented in Table 1 according to fold changes after 1 Gy and 2 Gy irradiation. The results show that a total of 28 proteins were up-regulated and 18 proteins were down-regulated. Of the 28 up-regulated proteins, 6 showed a fold change >2 (TSPYL2, FDXR, GMPR2, ACTN1, BAX and DDB2) for both doses. Of the 18 down-regulated proteins, 3 proteins (HBA1, HBB and HMGN4) showed a high response to radiation with a fold change >2. To test the dose dependent response, the correlation between protein expression and irradiated dose was evaluated for all 46 radiation responsive proteins. Proteins with high correlation (|correlation coefficient| > 0.70; *p* value < 0.05) are shown in Supplementary Table S3. The results show that the top 6 upregulated proteins (TSPYL2, FDXR, GMPR2, ACTN1, BAX and DDB2) have high dose-dependent response to radiation. Humanized mice used in this study were generated by injecting five different human donors’ stem cells. Figure 3 shows the dose response curves for FDXR, BAX, DDB2 and ACTN1 from 5 different human donors, 3 days after irradiation. These 4 proteins showed a dose-dependent response in human cells derived from each human donor. GMPR2 and TSPYL2 were not explored further as candidate biomarkers due to an incomplete data set caused by missing values and high variability between the donors. Although the top 3 down-regulated proteins (HBA1, HBB, HMGN4) showed fold changes >2, HBA1 and HBB had high variation between donors and HMGN4 did not show a dose-dependent response.

**Table 1:** List of differentially expressed proteins *in vivo*.

Accession no.	Protein name (Gene symbol)	Fold change^a^		
		1 Gy	2 Gy	adjusted *p* value^b^
**Upregulated proteins**				
Q9H2G4	Testis-specific Y-encoded-like protein 2 (TSPYL2)	14.97 ± 5.50	15.26 ± 5.69	3.81E-12^c^
P22570	NADPH:adrenodoxin oxidoreductase, mitochondrial (FDXR)	2.85 ± 0.23	3.47 ± 0.33	6.70E-11^c^
H0YNJ6	GMP reductase (GMPR2)	2.23 ± 0.16	2.59 ± 0.17	1.94E-09^c^
P12814	Alpha-actinin-1 (ACTN1)	2.03 ± 0.13	2.56 ± 0.14	1.01E-09^c^
Q07812	Apoptosis regulator BAX (BAX)	2.32 ± 0.28	2.37 ± 0.17	8.96E-09^c^
Q92466	DNA damage-binding protein 2 (DDB2)	2.04 ± 0.20	2.21 ± 0.18	1.12E-08^c^
Q8WWP7	GTPase IMAP family member 1 (GIMAP1)	1.89 ± 0.23	2.02 ± 0.33	1.39E-08^c^
Q6UXH1	Cysteine-rich with EGF-like domain protein 2 (CRELD2)	1.62 ± 0.10	2.01 ± 0.26	1.73E-08^c^
Q9NRX4	14 kDaphosphohistidine phosphatase (PHPT1)	1.77 ± 0.19	1.93 ± 0.22	2.73E-09^e^
P06127	T-cell surface glycoprotein CD5 (CD5)	1.75 ± 0.12	1.82 ± 0.23	1.43E-08^c^
P04083	Annexin A1 (ANXA1)	1.20 ± 0.06	1.73 ± 0.15	2.44E-08^e^
Q01831	DNA repair protein complementing XP-C cells (XPC)	1.29 ± 0.10	1.58 ± 0.12	2.19E-07^e^
P21291	Cysteine and glycine-rich protein 1 (CSRP1)	1.49 ± 0.10	1.52 ± 0.14	4.73E-07^e^
P17612	cAMP-dependent protein kinase catalytic subunit alpha (PRKACA)	1.28 ± 0.09	1.52 ± 0.02	2.28E-09^c^
A0A087WZM2	Ribonuclease T2 (RNASET2)	1.26 ± 0.05	1.48 ± 0.10	2.98E-09^c^
P08311	Cathepsin G (CTSG)	1.64 ± 0.20	1.44 ± 0.13	2.71E-08^d^
Q9UHD8	Septin-9 (SEPT9)	1.22 ± 0.06	1.36 ± 0.07	4.09E-08^c^
Q96HC4	PDZ and LIM domain protein 5 (PDLIM5)	1.21 ± 0.09	1.34 ± 0.08	1.73E-08^e^
P19367	Hexokinase-1 (HK1)	1.09 ± 0.05	1.34 ± 0.09	2.02E-08^c^
P08133	Annexin A6 (ANXA6)	1.15 ± 0.06	1.30 ± 0.05	4.20E-08^c^
P21283	V-type proton ATPase subunit C 1 (ATP6V1C1)	1.26 ± 0.09	1.29 ± 0.07	1.74E-07^e^
P48426	Phosphatidylinositol 5-phosphate 4-kinase type-2 alpha (PIP4K2A)	1.30 ± 0.06	1.25 ± 0.05	1.04E-07^d^
P20073	Annexin A7 (ANXA7)	1.15 ± 0.06	1.24 ± 0.07	1.12E-07^e^
P46777	60 S ribosomal protein L5 (RPL5)	1.09 ± 0.13	1.23 ± 0.18	1.46E-06^e^
O75083	WD repeat-containing protein 1 (WDR1)	1.14 ± 0.05	1.21 ± 0.07	1.98E-08^c^
Q9Y490	Talin-1 (TLN1)	1.20 ± 0.05	1.20 ± 0.06	5.18E-08^c^
A0A024R4M0	40 S ribosomal protein S9 (RPS9)	1.30 ± 0.11	1.19 ± 0.11	3.00E-08^d^
A0A0A0MT22	Protein tyrosine phosphatase, receptor type, C, isoform CRA_d (PTPRC)	1.22 ± 0.05	1.10 ± 0.07	1.24E-07^d^
**Down-regulated proteins**				
P69905	Hemoglobin subunit alpha (HBA1)	10.91 ± 3.30	12.60 ± 2.94	4.44E-10^c^
P68871	Hemoglobin subunit beta (HBB)	9.64 ± 0.14	10.63 ± 1.31	4.92E-10^c^
O00479	High mobility group nucleosome-binding domain-containing protein 4 (HMGN4)	2.25 ± 0.14	2.16 ± 0.32	8.23E-08^c^
E7EX17	Eukaryotic translation initiation factor 4B (EIF4B)	2.00 ± 0.19	1.89 ± 0.20	1.74E-08^c^
Q9Y2W1	Thyroid hormone receptor-associated protein 3 (THRAP3)	1.92 ± 0.21	1.83 ± 0.14	3.83E-11^c^
P62263	40 S ribosomal protein S14 (RPS14)	1.80 ± 0.29	1.82 ± 0.23	8.72E-08^c^
Q9NYF8	Bcl-2-associated transcription factor 1 (BCLAF1)	1.56 ± 0.10	1.82 ± 0.18	7.39E-09^c^
O15400	Syntaxin-7 (STX7)	1.76 ± 0.23	1.65 ± 0.16	3.34E-08^c^
D6RBZ0	Heterogeneous nuclear ribonucleoprotein A/B (HNRNPAB)	1.61 ± 0.12	1.62 ± 0.05	1.94E-11^c^
P38159	RNA-binding motif protein, X chromosome (RBMX)	1.56 ± 0.14	1.57 ± 0.09	6.49E-09^c^
P35611	Alpha-adducin (ADD1)	1.48 ± 0.11	1.46 ± 0.05	9.65E-08^c^
Q96PK6	RNA-binding protein 14 (RBM14)	1.36 ± 0.07	1.43 ± 0.06	4.91E-10^c^
O75400	Pre-mRNA-processing factor 40 homolog A (PRPF40A)	1.37 ± 0.07	1.42 ± 0.07	9.52E-09^c^
Q00839	Heterogeneous nuclear ribonucleoprotein U (HNRNPU)	1.28 ± 0.05	1.40 ± 0.08	4.29E-09^c^
X6R4W8	BUB3-interacting and GLEBS motif-containing protein ZNF207 (ZNF207)	1.25 ± 0.10	1.39 ± 0.13	1.57E-08^c^
Q12874	Splicing factor 3 A subunit 3 (SF3A3)	1.22 ± 0.04	1.38 ± 0.09	1.27E-08^c^
Q13435	Splicing factor 3B subunit 2 (SF3B2)	1.24 ± 0.08	1.32 ± 0.08	3.35E-08^c^
Q9NR30	Nucleolar RNA helicase 2 (DDX21)	1.17 ± 0.03	1.29 ± 0.04	1.01E-08^c^

^a^Fold change compared to non-irradiated group was calculated and data represent mean ± SEM.

^b^Data were analysed using one-way ANOVA test. The details are included in Supplementary Table S2.

^c^*p* value obtained from comparison of three dose groups (0, 1 and 2 Gy) was described.

^d^*p* value obtained from comparison between 0 Gy vs. 1 Gy was described.

^e^*p* value obtained from comparison between 0 Gy vs. 2 Gy was described.

### Dose Prediction of candidate protein markers

The top 4 upregulated proteins (FDXR, BAX, DDB2 and ACTN1) were selected as the best candidates for dose predictions. Various combinations of these proteins were used to predict radiation dose by linear regression. In other words, levels of one or more of these proteins were used as predictor variables, whereas radiation dose was the outcome variable. Four models had substantial information theoretic support from the data, based on Akaike information criterion scores with sample size correction (AICc). They are shown in Table 2 and Supplementary Table S4. Those three models that included combinations of FDXR, ACTN1 and DDB2 had the strongest information theoretic support, lowest mean absolute errors (≤0.13 Gy) and highest coefficients of determination (R^2^ = 0.96) (Supplementary Table S4). The ability of each of these multi-protein models to predict dose is shown in Table 2, and the performance of various models containing only one protein is shown in Supplementary Table S5. These results suggest that FDXR and ACTN1 had strong associations with radiation dose (for each of them the sum of Akaike weights across all tested models was 1.0) and represent potentially valuable radiation biomarkers. DDB2 had a somewhat weaker association with dose (sum of Akaike weights was 0.14) but may still be a useful predictor.

**Table 2:** Dose prediction of candidate protein markers.

Model^a^	Predicted dose (Mean ± SD, Gy)			coefficient ^b^	R^2^ (*p* value)^b^	MAE^c^
	Actual irradiated dose					
	0 Gy	1 Gy	2 Gy			
Dose ~ FDXR + ACTN1	0.003 ± 0.050	1.06 ± 0.17	1.90 ± 0.24	0.95 ± 0.06	0.958 (<0.0001)	0.13
Dose ~ FDXR + ACTN1 + DDB2	0.001 ± 0.034	1.06 ± 0.14	1.91 ± 0.26	0.96 ± 0.06	0.959 (<0.0001)	0.12
Dose ~ FDXR + ACTN1 + BAX	0.001 ± 0.046	1.07 ± 0.16	1.90 ± 0.23	0.95 ± 0.06	0.958 (<0.0001)	0.13
Dose ~ FDXR + ACTN1 + DDB2 + BAX	0.13 ± 0.20	1.05 ± 0.14	1.91 ± 0.26	0.90 ± 0.06	0.942 (<0.0001)	0.15

^a^Data obtained from proteomic analysis were used for investigating dose prediction. Ranks and specific values of all models were provided in Supplementary Table S4.

^b^Coefficient, R^2^ and *p* value was obtained by linear regression analysis for predicted vs. actual doses.

^c^MAE values were used as indicators to compare the difference between actual irradiated and predicted dose.

### Verification of candidate protein markers in human lymphocytes *in vivo/in vitro*

**Figure 4:**
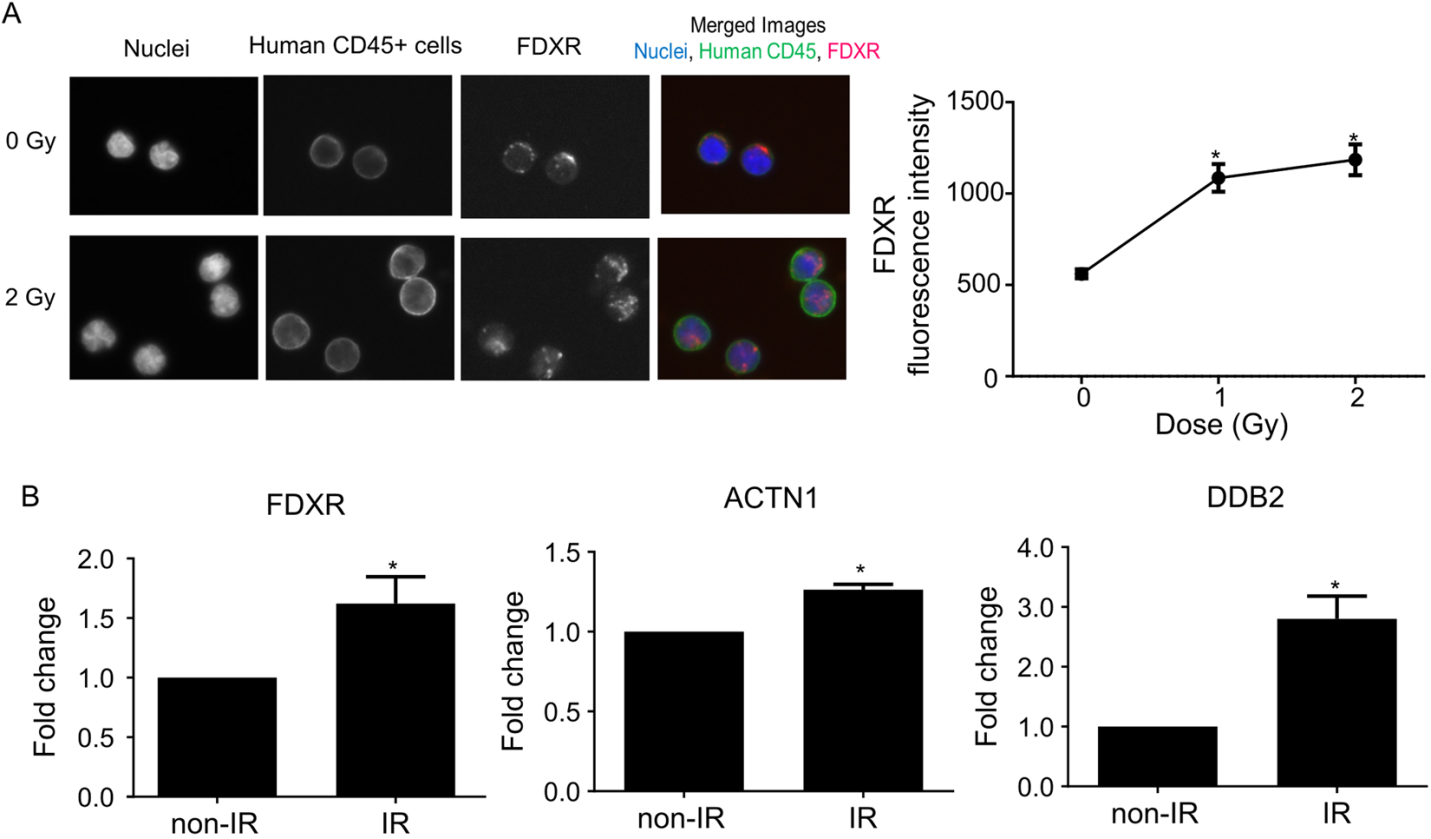
Verification of candidate protein expression in human lymphocytes. (**A**) Protein expression levels in human lymphocytes *in vivo*. Left panel shows representative images of FDXR stained human CD45+ lymphocytes after exposure to X rays (0 and 2 Gy). Right panel shows quantified FDXR expression level in human CD45+ cells from humanized mouse blood. Fluorescence intensity of FDXR was quantified and was analysed by Kruskall-Wallis test and Dunn’s post hoc multiple comparison test. Data indicate mean ± SEM. Asterisk (*) means statistically significant difference from control group (*p* value < 0.05). (**B**) Protein expression levels in human lymphocytes *in vitro*. FDXR, DDB2 and ACTN1 protein expression levels were measured in human lymphocytes cultured for 3 days after irradiation (0 and 4 Gy). Fold change of each protein level was calculated based on control. Data indicate mean ± SEM and analysed by Student’s t-test. Asterisk (*) means statistically significant difference from non-IR group (*p* value < 0.05).

Radiation-induced FDXR protein expression levels were evaluated in human CD45 + cells *in vivo* from the peripheral blood of the humanized mice (Fig. 4A). Representative images of FDXR protein observed in human CD45+ lymphocytes are shown after 2 Gy X-irradiation (Left panel in Fig. 4A). The results show a statistically significant increase in total FDXR fluorescence intensity in the 1 Gy and 2 Gy irradiated groups compared to the non-irradiated group (*p* value < 0.05; Right panel in Fig. 4A). Biomarker validation studies were also performed using human peripheral blood samples collected from up to 3-4 healthy human volunteers. The results show a significant increase in FDXR, ACTN1 and DDB2 protein levels in isolated human lymphocytes *in vitro*, 3 days after irradiation (Fig. 4B).

## Discussion

The identification of radiation-responsive proteins in blood samples that can rapidly and accurately quantify radiation exposure up to a week post irradiation will be critical for the mass-screening of individuals after a large-scale radiological event. In this scenario it is unlikely that blood samples will be collected from all potentially exposed individuals within 24–48 h. There is no perfect model for radiation biodosimetry and biomarker development: radiotherapy cancer patients are limited in terms of fixed fractionation doses and confounders of chemotherapy and underlying disease whereas the large cost of NHPs largely precludes developmental and systematic studies for many research groups. As an alternative model, we have used the hematopoietically Hu-NSG mouse model to identify radiation-responsive protein biomarkers in human lymphocytes *in vivo*, 3 days after radiation exposure.

Mass spectrometry-based proteomics analysis has been used routinely for biomarker discovery in clinical research. Applications of proteome profiling in radiation research are increasing, particularly as the research aims to study not only protein expression, but also function, structure, modifications and interactions^24^. Recently, proteomic changes in plasma and urine after irradiation of NHPs have also been studied^14,15^. In this study, we used global proteomics analysis to identify novel radiation biomarkers in X-irradiated human lymphocytes isolated from the Hu-NSG mouse spleen. We found a distinct protein signature 3 days post radiation exposure compared to the non-irradiated control group (Fig. 2). In total, 3,376 human proteins were identified of which 46 proteins were differentially expressed after radiation exposure. Twenty eight proteins were found up-regulated and 18 proteins were found down-regulated (Table 1), which showed distinct separation between non-irradiated group and irradiated groups by supervised PCA (Fig. 2). Of the 46 radiation-responsive proteins, four up-regulated proteins FDXR, BAX, DDB2 and ACTN1 were considered as the top 4 candidates based on large fold changes >2 and a strong correlation between protein expression and irradiated dose (correlation coefficient >0.7; *p* value < 0.05). Increased expression of the candidate proteins from the irradiated humanized mice groups derived from different human stem cell donors showed a reliable dose dependent response to radiation exposure (Fig. 3), which supports the finding that the radiation-induced response of our top candidates might have low inter-individual variation.

The top four candidates identified here are known to be associated with radiation-induced apoptosis, DNA damage and cellular senescence. FDXR is a mitochondrial flavoprotein transferring electron from NADPH to cytochrome p450. It can be induced by DNA damage and is involved in p53 and oxidative stress-mediated apoptosis^25,26^. In transcriptomics studies, FDXR gene is known as one of the most radiation responsive markers^27,28^ and has been validated as a sensitive gene for dose assessment in two large scale studies on laboratory inter-comparison of gene expression^5,29,30^. The FDXR gene has also shown a significant dose response over a large range of radiation doses in *ex vivo* X-irradiated human blood and radiotherapy treated patients and discriminated pre-irradiated and 24 h post-irradiated samples from prostate cancer patients^29,31,32^. BAX is a well-known regulator in radiation-induced apoptosis^33^. Radiation exposure activates BAX protein through ATM and CHK2 mediated p53 activation, leading to cytochrome c release and apoptotic cell death^34^. DDB2 is a protein associated with nucleotide excision repair and has a key role in DNA damage recognition. It facilitates ATM/ATR recruitment to damage sites and leads to apoptosis by regulating p21 protein^35^. ACTN1 is an actin binding protein regulating actin cytoskeleton^36^, and is one of the cellular senescence related proteins^37,38^. Chronic radiation exposure has been shown to cause premature senescence with an increase of ACTN1 in human umbilical vein endothelial cells^39^.

Our results (Table 2 and Supplementary Table S4) showed that combinations of FDXR, DDB2 and ACTN1 proteins generate an accurate prediction of the radiation dose in human lymphocytes *in vivo*: their mean absolute errors were ≤0.13 Gy over the tested dose range of 0 to 2 Gy. Previous studies have similarly used a combinatorial approach of plasma protein markers to provide an improved dose assessment compared to a single biomarker alone in mouse^10^ and NHP models^13^. Budworth *et al*.^40^ showed that a 8-gene transcript panel of DNA repair markers (*BBC3*, *FDXR*, *CDKN1A*, *GADD45a*, *PCNA*, *XPC*, *DDB2 and POLH*) was able to discriminate radiation exposure in the human *ex vivo* blood model and *in vivo* irradiated human blood samples obtained from patients who received total body irradiation, 24 h after irradiation^40^, whereas Manning and colleagues^31^, combined *FDXR* and *DDB2* gene expression response to produce the best dose estimate in human blood cells 24 h after X-irradiation (1 and 2 Gy). The notable finding in this study is that we used our 3-protein panel to predict dose 3 days after radiation. This has the potential to extend the window of time relevant for medical decision making for radiological triage.

Biomarker validation studies were performed to evaluate the radiation-induced response using indirect immunofluorescence assay protocols. Using available Hu-NSG peripheral blood samples, our initial validation studies focused on the FDXR protein biomarker. Consistent with the results of proteomic analysis in human CD45+ lymphocytes isolated from the mouse spleen, we observed a similar dose-dependent increase of total FDXR protein fluorescence in human CD45+ lymphocytes obtained from the peripheral mouse blood after X-ray exposure (Fig. 4A). Future studies using an independent validation data set for each biodosimetry marker will confirm the capability of our protein panel to accurately predict dose. Several studies to date have validated FDXR transcriptional gene expression in blood leukocytes *ex vivo*^40,41^ as a biomarker of radiation exposure, however few studies have validated this biomarker *in vivo*^29,32^. Recently, O’Brien *et al*.^32^ carried out an extensive study and provided for the first time physical and biological dose estimates using FDXR gene expression in human leukocytes from radiotherapy patients *in vivo*.

Previous studies by Amundson and others^27,42,43^ have successfully used the human *ex vivo* model to closely replicate the *in vivo* gene expression response to radiation. To assess radiation-induced response of protein markers in human lymphocytes irradiated *ex vivo*, we measured expression levels of our three best candidates, FDXR, DDB2 and ACTN1 protein, 3 days after initial exposure (Fig. 4B). Since NSG mice are relatively sensitive with a LD50/30 of ~ 3–4 Gy^23^, we irradiated human peripheral blood with a dose of 4 Gy γ rays *ex vivo* to represent the sensitivity of the Hu-NSG mouse response at 2 Gy. The results show an increase in expression of FDXR, DDB2 and ACTN1, which allowed us to discriminate between non-irradiated and irradiated samples. Although there was no apparent large variation in biomarker response between human donors recruited here, it will be important to plan a population study in the future, to evaluate our protein panel and other top up and down regulated biomarkers for the effects of individual factors (age, gender, smoking history etc.) after radiation exposure.

It is well-known that irradiated human lymphocytes undergo apoptosis in a time- and dose- dependent manner^44–46^. Contrary to the *in vivo* model, where dying cells appear to be cleared from the circulation by the liver, the human blood *ex vivo* model represents a different microenvironment whereby biological processes and related measurements could be affected by artifacts such as increased DNA fragmentation and apoptosis, that could be a confounding factor in the day 3 cultures. To this end, future studies should aim to evaluate the dose-response relationship with extended doses and time kinetics for the top radio-responsive proteins and statistically compare the day 3 fold-changes with earlier and later time points up to a week post-exposure. Previous studies to date, have reported increased gene/protein expression of our candidate biomarkers 24–48 h after radiation exposure^32,40,41,47^, whereas less is known about the longer time points. In the present work, we have combined the humanized mouse model and the human *ex vivo* model to investigate candidate protein biomarkers in human blood leukocytes after acute radiation exposure. Future studies designed to verify and validate the candidate proteins identified here, provide the opportunity to develop a biodosimetry assay for use in rapid, early triage following a large scale radiological incident or accident.

## Summary

To our knowledge, this is the first study that has used the hematopoietically Hu-NSG model for biological radiation dosimetry studies. Results show that the Hu-NSG mice were successfully engrafted with HSCs to produce human hematopoietic progenitor and differentiated cells for proteomic study. Our goal was to identify protein markers for radiation biodosimetry 3 days after radiation exposure *in vivo*. Three proteins (FDXR, DDB2 and ACTN1) were determined as the best candidate markers and combined expression of these three proteins predicted more accurately the absorbed dose than either one alone. The fact that we have identified known radiation-responsive proteins *in vivo* as candidate biomarkers in human lymphocytes to accurately predict radiation dose, 3 days after acute radiation exposure supports the use of the humanized mouse model for the development of biodosimeters for use in radiation triage. Further studies are needed to optimize and validate our biomarker panel to estimate retrospective dose up to a week after exposure and to advance high-throughput screening methods for the rapid immunodetection of radiation-responsive biomarkers in blood samples for radiological triage.

## Methods

All methods were carried out in accordance with relevant guidelines and regulations.

### Humanized mice model

All animal experiments were approved by the Columbia University Institutional Animal Care and Use Committee (IACUC; approved protocol AAP9613) and were conducted under all relevant federal and state guidelines. Female immunodeficient NOD.Cg-*Prkdc*^*scid*^ *Il2rg*^*tm1Wjl*^/SzJ (NSG) mice (The Jackson Laboratory; Bar Harbor, ME, USA), aged 6 to 8 weeks, were engrafted with commercially available human cord blood CD34+ cells (Cincinnati Children’s Hospital Medical Center; Cincinnati, OH, USA). For the engraftment, the NSG mice were irradiated with 2.0 Gy of gamma rays followed by injection of 200,000 human CD34+ cells (n = 5 human donors) in the mouse tail vein within 24 hours after the irradiation. Human cell engraftment was determined four months later by flow cytometry using 20 μl of mouse tail vein blood, stained with antibodies specific to human CD45, (WBC marker, clone HI30, Biolegend, San Diego, CA), human CD3 (T cell marker, clone UCHT1; Biolegend), human CD20 (B cell marker, clone 2H7; Biolegend) and mouse CD45, (WBC marker, clone 30-F11, eBioscience, San Diego, CA). The study was designed using two cohorts of humanized mice engrafted with 5 different donors of human CD34+ cells. Cohort 1 (16 mice) were injected with donors #1–3 and Cohort 2 (17 mice) with donors #4–5.

### Irradiation

The humanized mice used here are relatively radiosensitive with a LD50 of about 3~4Gy^23^. As a result, this proteomics study was designed for 1 Gy and 2 Gy doses *in vivo* model. X-irradiation was performed using X-RAD 320 biological irradiator (Precision X-ray, North Branford, CT) operating at 320Kv, 12.5 mA. Total 33 humanized mice were randomly grouped and irradiated with 0, 1 and 2 Gy (0 Gy, n = 7; 1 Gy, n = 11; 2 Gy, n = 15) at a dose rate of 88 cGy/min. Spleen and blood samples were collected 3 days after irradiation for proteomic analysis and immunofluorescence assay.

### Sample preparation for proteomic analysis

Spleens were homogenized and filtered through 40μm nylon strainer to obtain a single cell suspension. Cells were resuspended in autoMACS Rinsing Solution (Miltenyi Biotec, Bergisch Gladbach, Germany) with MACS BSA stock solution (Miltenyi Biotec). Small portion of cells were first stained with human CD45 clone HI30, human CD3 clone UCHT1, human CD20 clone 2H7, and mouse CD45 clone 30-F11. Cell numbers in each spleen sample were measured with LSR Fortessa Cell Analyzer (BD Biosciences, San Jose, CA) and data were analysed with FlowJo software (Tree Star, Inc., San Carlos, CA). Based on the cell number, whole spleen cell suspension was incubated with human CD45 antibody (Clone HI30). Human CD45+ cells were collected in PBS by FACS Aria (BD Biosciences). To obtain enough cell number for proteomic analysis, samples were pooled based on the number of collected cells by flow cytometry. After pooling samples, total 21samples (0 Gy, n = 7; 1 Gy, n = 7; 2 Gy, n = 7) were prepared for proteomic analysis. Cell pellets were frozen and stored at −80 °C until use.

### Protein Extraction and Digestion

Each cell pellet was homogenized in lysis buffer (4 M Urea in 50 mM Ammonium Bicarbonate, 0.1% RapiGest^TM^; Waters, MA) and Roche protease inhibitor cocktail) and protein concentration was measured by the Qubit Protein Quantification Assay (Thermo Fisher Scientific, Rockford, IL). An equal amount of protein lysate from each sample was pooled as a common reference to generate ratios in the TMT10plex labeling experiment. 15 μg of protein lysates were reduced with 10 mM TCEP and alkylated with iodoacetamide (18.75 mM) in the dark for 30 minute. Protein was precipitated by the methanol-chloroform protocol and re-suspended in 100 mM TEAB. 375 ng of trypsin was added to the protein extract in a 1:40 enzyme to protein ratio and incubated for 16 hours at 37 °C. Next day, samples were centrifuged for 30 min at 14,000 rpm to remove insoluble materials and 0.6 μg of the peptide mixture was used for peptide quantification assay (Thermo Fisher Scientific).

### Tandem Mass Tagging Labeling

Isobaric labeling of digested protein lysates was performed using the 10-plex tandem mass tag (TMT) reagents (Thermo Fisher Scientific). TMT reagents (0.8 mg) were dissolved in 41 μl of dry acetonitrile (ACN), and 7.5 μl was added to 15 μg of digested lysate in 100 mM TEAB. After 1 hour incubation at room temperature, the reaction was quenched by adding 3 μl of 5% hydroxylamine. Labeled peptides were combined and dried for the subsequent high pH reverse phase peptide fractionation (Thermo Fisher Scientific). Eight fractions were generated for each combined set.

### LC-MS/MS Analysis

Each TMT labeled fraction was reconstituted in a solution of 2%ACN, 2% formic acid (FA) for MS analysis. Peptides were eluted from the easy-spray column (50 cm) using a Dionex Ultimate 3000 Nano LC system with a 30 min gradient from 5% buffer B to 20% buffer B (100% ACN, 0.1% FA) and 10 min gradient from 20%B to 30%B. The gradient was switched from 30% to 85% buffer B over 1 min and held constant for 3 min. Finally, the gradient was changed from 85% buffer B to 98% buffer A (100% water, 0.1% FA) over 1 min, and then held constant at 98% buffer A for 20 minutes. The application of a 2.0 kV distal voltage electrosprayed the eluting peptides directly into the Thermo Fusion Tribrid mass spectrometer equipped with an EASY-Spray source (Thermo Fisher Scientific). Mass spectrometer-scanning functions and HPLC gradients were controlled by the Xcalibur data system (Thermo Finnigan, San Jose, CA).

For all experiments, the instrument was operated in the data-dependent mode. We collected FTMS1 spectra at a resolution of 120 K with an automated gain control (AGC) 400,000, a max injection time of 50 ms. The 10 most intense ions were selected for MS/MS. Precursors were filtered according to charge state (z = 2–7), and monoisotopic peak assignment. Previously interrogated precursors were excluded using a dynamic exclusion duration of 60 s. For the FTMS3 method, ITMS2 spectra were collected at AGC of 100,000, max injection time of 105 ms, and CID collision energy of 35%. FTMS3 spectra utilized the same Orbitrap parameters as FTMS2 method, except HCD collision energy was increased to 55% to ensure maximal TMT report ion yield. Synchronous-precursor-selection (SPS) was enabled to include up to 3, 6, or 10 MS2 fragment ions in the FTMS3 scan.

### Database Search and Assignment of MS/MS Spectra

Tandem mass spectra from raw files were searched against a human protein database using the Proteome Discoverer 2.1 (Thermo Finnigan). The Proteome Discoverer application extracts relevant MS/MS spectra from the raw file and determines the precursor charge state and the quality of the fragmentation spectrum. The Proteome Discoverer probability-based scoring system rates the relevance of the best matches found by the SEQUEST algorithm. The human protein database was downloaded as FASTA-formatted sequences from UniProt protein database (released in October, 2016). The peptide mass search tolerance was set to 10ppm. A minimum sequence length of 7 amino acids residues was required. Only fully tryptic peptides were considered. To calculate confidence levels and FDR, Proteome Discoverer generates a decoy database containing reverse sequences of the non-decoy protein database and performs the search against this concatenated database (non-decoy + decoy). 5% FDR was used to generate the quantitative list for statistical analysis.

### Determination of TMT Reporter Ion Intensities and Quantitative Data Analysis

For quantification, a 0.003 m/z (10plex TMT) window centered on the theoretical m/z value of each reporter ion was queried for the nearest signal intensity. Reporter ion intensities were adjusted for the isotopic impurities of each TMT reagent according to the manufacture specifications. The signal-to-noise (S/N) values for all peptides were summed within each TMT channel, and each channel was scaled according to the inter channel difference of these sums to account for differences in sample handling. For each peptide, a total minimum sum S/N of 10 and an isolation purity greater than 75% was required. Total peptide amount was used for normalization to correct for experimental bias. Using the Qlucore Omics Explorer 2.2 (Qlucore AB, Lund, Sweden), ANOVA was applied to generate a protein signature from each comparison. Multiple test correction was performed by adjusting the calculated *p* values according to Benjamini-Hochberg method.

### Immunofluorescence assay in humanized mouse model

Peripheral mononuclear cells (PBMCs) were isolated from blood samples of humanized mice (generated from 2 different human stem cell donors) by the Ficoll density gradient method. Whole blood samples were diluted to RPMI medium (Gibco, Waltham, MA), layered on Histopaque1083 (Sigma-Aldrich, St. Louis, MO), and centrifuged at 1220 rpm for 45 min. The buffy coat of PBMCs was taken and washed with PBS. To label human CD45+ cells, the isolated PBMC cells were incubated with rat anti-human CD45 (Thermo Fisher Scientific) for 30 min and fixed with 2% paraformaldehyde (Electron Microscopy Sciences, Hatfield, PA) for 20 min. Cells were washed with PBS and cytospun (Cytospin 4; Thermo Scientific, Waltham, Boston, MA) onto coated microscope slides (CYTOSLIDE™; Thermo Scientific) and then placed into PBS. For candidate biomarker staining, cells on the slides were treated with 0.5% Triton-X 100, blocked with 3% bovine serum albumin (Sigma-Aldrich) and incubated with the following antibodies: rabbit anti-FDXR (Sigma-Aldrich), Alexa fluor 555 goat anti-rabbit IgG (Thermo Fisher Scientific) and Alexa fluor 488 goat anti-rat IgG (Thermo Fisher Scientific). Cells were counterstained and mounted with VECTASHIELD Antifade Mounting Medium with DAPI (Vector laboratories, Burlingame, CA). Quantification of FDXR protein levels was performed using image analysis software described previously^48^ with the following modification: human lymphocytes were identified and selected from binarized images for human CD45 using Otsu’s algorithm^49^. Fluorescence values from a second image, corresponding to the immunostaining for FDXR, were integrated over the cellular area. Fluorescence intensity values were background subtracted, normalized to the cellular area and reported as bits/pixel.

### Immunofluorescence assay in human lymphocytes *in vitro*

Research was approved by the Columbia University Medical Center Institutional Review Board (IRB-AAAF2671) and informed written consent was obtained from all participants. Peripheral whole blood samples from healthy human volunteers were collected in heparin tube and irradiated with γ rays (0 and 4 Gy) using a Gammacell 40 ^137^Cs irradiator (Atomic Energy of Canada, Ltd.). Peripheral blood was incubated in RPMI 1640 (Gibco) with 15% FBS and 2% Penicillin and Streptomycin at 37 °C. PBMCs were isolated from cultured blood samples (n = 3 ~ 4) 3 days after radiation exposure and were prepared for immunofluorescent labeling using the same protocol described above in the humanized mice model. Cells were incubated with the following antibodies: rabbit anti-FDXR, rabbit anti-DDB2 (Thermo Fisher Scientific), rabbit anti-ACTN1 (Cell Signaling Technology, Danvers, MA), and Alexa fluor 488 goat anti-rabbit IgG. Fluorescence levels of candidate proteins and percentages of positive cells based on maximum pixel intensity were measured and fold changes compared to baseline level were calculated. Quantification of radiation-induced protein expression levels was determined in non-apoptotic cells only by the image analysis software program^48^, such that advanced apoptotic cells observed as a gross change in area and morphology of the DAPI-labeled nuclei, were not included for analysis.

### Statistical analysis

Statistical analysis was performed using R software (version 3.4.0; https://www.r-project.org/) and GraphPad Prism (version 6.01; GraphPad Software, Inc., La Jolla, CA). Protein expression levels of radiation responsive proteins identified in proteomic analysis were further analysed to find best candidate markers and panels. Correlation between irradiated dose and protein abundance were analysed by Pearson correlation coefficients. Multiple linear regression analysis was used to develop dose-response relationship for protein combination for dose assessment. The best fitted model for each parameter was applied to pooled dataset to estimate irradiation dose. We used Akaike Information Criterion (AIC)-based method to compare all single/combination models. AICc score, a corrected value of AIC for small sample size, and Akaike weight for each model was computed to determine the best model for dose prediction. The capability of candidates to predict dose was compared using mean absolute error (MAE) of predicted dose vs. actual dose. Possible influence by different experimental batches or cohorts were controlled by statistical analysis using batch variables or normalizing data with Succinate dehydrogenase [ubiquinone] flavoprotein subunit (SDHA), one of endogenous controls. There was no significant difference in SDHA level between different dose groups, and SDHA normalization did not affect the results obtained from original dataset. FDXR fluorescence intensity was compared by Kruskall-Wallis test and Dunn’s post hoc test. Fold changes of each candidate protein were compared by Student’s t-test. Two-tailed *p* values less than 0.05 were considered statistically significant.

## Electronic supplementary material

Supplementary information

Supplementary Dataset 1

## Data Availability

All data generated or analysed during this study are included in this published article and its Supplementary Dataset & Information files.
