# Supplementary material for: Candidate protein markers for radiation biodosimetry in the hematopoietically humanized mouse model
Source: Sci Rep. 2018 Sep 10;8:13557. doi: 10.1038/s41598-018-31740-8 (PMC6131502; doi:10.1038/s41598-018-31740-8)
Supplement: Supplementary file 1 — Supplementary information [file 41598_2018_31740_MOESM1_ESM.pdf]

Supplementary information

Candidate protein markers for radiation biodosimetry in the hematopoietically humanized mouse model

Younghyun Lee[1,*], Monica Pujol Canadell[1], Igor Shuryak[1], Jay R. Perrier[1], Maria Taveras[1], Purvi Patel[2], Antonius Koller[2], Lubomir B. Smilenov[1], David J. Brenner[1], Emily I. Chen[2,3], Helen C. Turner[1,*]

[1]Center for Radiological Research, Columbia University Medical Center, New York, NY 10032, USA
[2]Herbert Irving Comprehensive Cancer Center, Proteomics Shared Resource, Columbia University Medical Center, New York, NY 10032, USA
[3]Department of pharmacology, Columbia University Medical Center, New York, NY 10032, USA

* Corresponding Authors;

Younghyun Lee
Center for Radiological Research, Columbia University Medical Center, 630 West 168th St, VC11-239, New York, NY 10032, USA
Phone: 1-212-342-6838
Email: younghyun.lee.0123@gmail.com

Helen C. Turner
Center for Radiological Research, Columbia University Medical Center, 630 West 168th St, VC11-234, New York, NY 10032, USA
Phone: 1-212-305-6058
Email: HT2231@cumc.columbia.edu

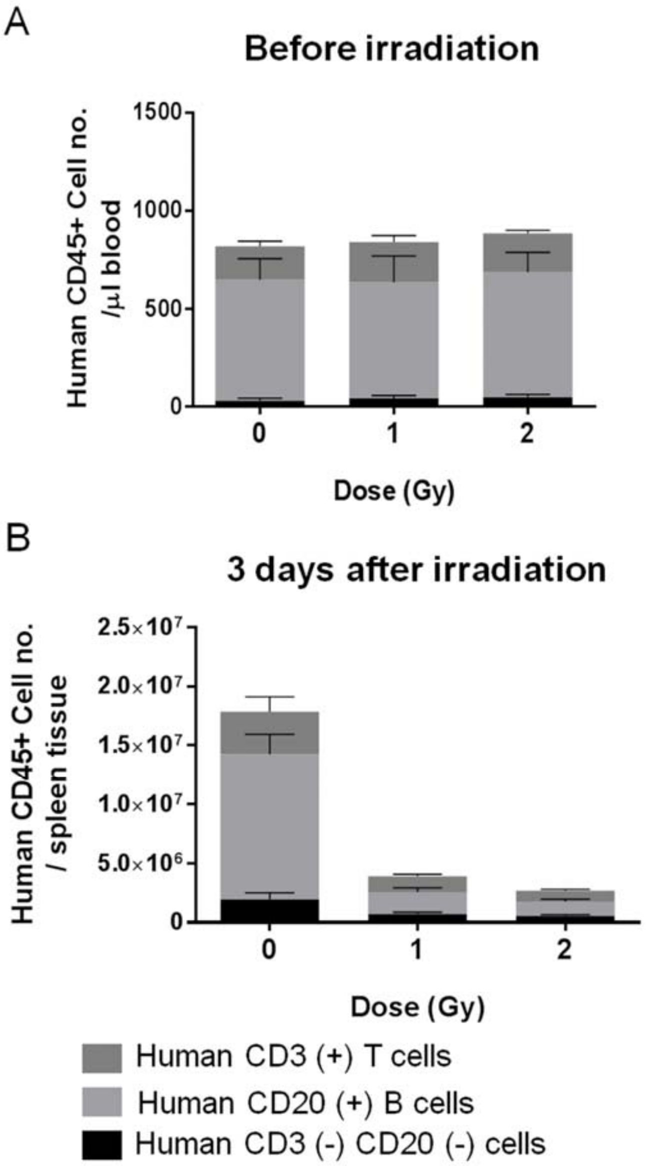

Supplementary Figure S1. The number of human lymphocyte (A) in humanized mouse blood before irradiation and (B) in spleen tissues 3 days after irradiation. Data represent mean ± SEM.

Supplementary Table S1. The number of human and mouse lymphocytes in tail blood of humanized mice before irradiation

Mouse ID	Dose Group (Gy)	Human Donor ID	Cell no. (no./μl blood)				Human CD45+ cells (%)[a]
			Human CD45+ cells	Human CD20+ cells	Human CD3+ cells	Mouse CD45+ cells	
Mouse #1	0	Donor #1	510	387	120	235	68
Mouse #2	0	Donor #2	1,059	800	280	406	72
Mouse #3	0	Donor #3	908	714	249	395	70
Mouse #4	0	Donor #3	491	404	108	154	76
Mouse #5	0	Donor #4	1,224	1,014	128	353	78
Mouse #6	0	Donor #4	1,041	796	169	258	80
Mouse #7	0	Donor #5	401	214	142	858	32
Mouse #8	1	Donor #1	577	443	165	403	59
Mouse #9	1	Donor #1	797	552	222	288	73
Mouse #10	1	Donor #2	1,213	869	428	697	64
Mouse #11	1	Donor #3	985	700	296	541	65
Mouse #12	1	Donor #3	664	504	187	387	63
Mouse #13	1	Donor #4	1,546	1,226	228	590	72
Mouse #14	1	Donor #4	1,942	1,476	304	439	82
Mouse #15	1	Donor #5	344	201	115	638	35
Mouse #16	1	Donor #5	546	283	202	623	47
Mouse #17	1	Donor #5	252	156	53	546	32
Mouse #18	1	Donor #5	225	111	61	912	20
Mouse #19	2	Donor #1	768	551	225	348	69
Mouse #20	2	Donor #1	772	559	238	226	77
Mouse #21	2	Donor #2	1,544	1,270	242	467	77
Mouse #22	2	Donor #2	668	582	151	567	54
Mouse #23	2	Donor #3	1,213	820	323	757	62
Mouse #24	2	Donor #3	515	383	129	529	49
Mouse #25	2	Donor #3	645	444	223	303	68
Mouse #26	2	Donor #4	1,298	894	205	583	69
Mouse #27	2	Donor #4	1,371	1,122	178	428	76
Mouse #28	2	Donor #4	1,360	1,056	207	371	79
Mouse #29	2	Donor #4	1,509	1,164	252	304	83
Mouse #30	2	Donor #5	214	94	72	951	18
Mouse #31	2	Donor #5	511	324	158	678	43
Mouse #32	2	Donor #5	251	103	130	232	52
Mouse #33	2	Donor #5	515	229	231	337	60

[a]percentage of human CD45+ cells in total cells including human and mouse cells

Supplementary Table S2. Identification of radiation responsive proteins

Accession no.	Protein name (Gene symbol)[a]	*p* value	*q* value
**Comparison of three dose groups (0, 1, and 2 Gy) at 0.001% FDR**			
A0A087WZM2	Ribonuclease T2 (RNASET2)	2.98E-09	9.15E-07
D6RBZ0	Heterogeneous nuclear ribonucleoprotein A/B (HNRNPAB)	1.94E-11	3.27E-08
E7EX17	Eukaryotic translation initiation factor 4B (EIF4B)	1.74E-08	2.45E-06
H0YNJ6	GMP reductase (GMPR2)	1.94E-09	7.27E-07
O00479	High mobility group nucleosome-binding domain-containing protein 4 (HMGN4)	8.23E-08	8.68E-06
O15400	Syntaxin-7 (STX7)	3.34E-08	4.04E-06
O75083	WD repeat-containing protein 1 (WDR1)	1.98E-08	2.62E-06
O75400	Pre-mRNA-processing factor 40 homolog A (PRPF40A)	9.52E-09	2.00E-06
P06127	T-cell surface glycoprotein CD5 (CD5)	1.43E-08	2.30E-06
P08133	Annexin A6 (ANXA6)	4.20E-08	4.73E-06
P12814	Alpha-actinin-1 (ACTN1)	1.01E-09	4.27E-07
P17612	cAMP-dependent protein kinase catalytic subunit alpha (PRKACA)	2.28E-09	7.69E-07
P19367	Hexokinase-1 (HK1)	2.02E-08	2.62E-06
P22570	NADPH:adrenodoxin oxidoreductase, mitochondrial (FDXR)	6.70E-11	5.65E-08
P35611	Alpha-adducin (ADD1)	9.65E-08	9.58E-06
P38159	RNA-binding motif protein, X chromosome (RBMX)	6.49E-09	1.69E-06
P62263	40S ribosomal protein S14 (RPS14)	8.72E-08	8.92E-06
P68871	Hemoglobin subunit beta (HBB)	4.92E-10	2.37E-07
P69905	Hemoglobin subunit alpha (HBA1)	4.44E-10	2.37E-07
Q00839	Heterogeneous nuclear ribonucleoprotein U (HNRPU)	4.29E-09	1.21E-06
Q07812	Apoptosis regulator BAX (BAX)	8.96E-09	2.00E-06
Q12874	Splicing factor 3A subunit 3 (SF3A3)	1.27E-08	2.26E-06
Q13435	Splicing factor 3B subunit 2 (SF3B2)	3.35E-08	4.04E-06
Q6UXH1	Cysteine-rich with EGF-like domain protein 2 (CRELD2)	1.73E-08	2.45E-06
Q8WWP7	GTPase IMAP family member 1 (GIMAP1)	1.39E-08	2.30E-06
Q92466	DNA damage-binding protein 2 (DDB2)	1.12E-08	2.09E-06
Q96PK6	RNA-binding protein 14 (RBM14)	4.91E-10	2.37E-07
Q9H2G4	Testis-specific Y-encoded-like protein 2 (TSPYL2)	3.81E-12	1.29E-08
Q9NR30	Nucleolar RNA helicase 2 (DDX21)	1.01E-08	2.00E-06
Q9NYF8	Bcl-2-associated transcription factor 1 (BCLAF1)	7.39E-09	1.78E-06
Q9UHD8	Septin-9 (SEPT9)	4.09E-08	4.73E-06
Q9Y2W1	Thyroid hormone receptor-associated protein 3 (THRAP3)	3.83E-11	4.31E-08
Q9Y490	Talin-1 (TLN1)	5.18E-08	5.64E-06
X6R4W8	BUB3-interacting and GLEBS motif-containing protein ZNF207 (ZNF207)	1.57E-08	2.41E-06
**Comparison between 0 Gy vs. 1 Gy at 0.010% FDR**			
A0A024R4M0	40S ribosomal protein S9 (RPS9)	3.00E-08	1.14E-05
A0A0A0MT22	Protein tyrosine phosphatase, receptor type, C, isoform CRA_d (PTPRC)	1.24E-07	2.34E-05
D6RBZ0	Heterogeneous nuclear ribonucleoprotein A/B (HNRNPAB)	1.28E-08	9.70E-06
O75083	WD repeat-containing protein 1 (WDR1)	2.77E-07	4.50E-05
P06127	T-cell surface glycoprotein CD5 (CD5)	3.29E-07	4.99E-05
P08311	Cathepsin G (CTSG)	2.71E-08	1.14E-05
P21291	Cysteine and glycine-rich protein 1 (CSRP1)	5.20E-08	1.48E-05

P22570	NADPH:adrenodoxin oxidoreductase, mitochondrial (FDXR)	9.89E-10	1.12E-06
P38159	RNA-binding motif protein, X chromosome (RBMX)	2.36E-08	1.14E-05
P48426	Phosphatidylinositol 5-phosphate 4-kinase type-2 alpha (PIP4K2A)	1.04E-07	2.16E-05
Q6UXH1	Cysteine-rich with EGF-like domain protein 2 (CRELD2)	5.28E-07	7.50E-05
Q8WWP7	GTPase IMAP family member 1 (GIMAP1)	6.63E-07	8.37E-05
Q92466	DNA damage-binding protein 2 (DDB2)	1.03E-07	2.16E-05
Q9H2G4	Testis-specific Y-encoded-like protein 2 (TSPYL2)	3.36E-17	7.65E-14
Q9NR30	Nucleolar RNA helicase 2 (DDX21)	6.05E-07	8.09E-05
Q9NRX4	14 kDaphosphohistidine phosphatase (PHPT1)	1.67E-07	2.93E-05
Q9Y2W1	Thyroid hormone receptor-associated protein 3 (THRAP3)	9.89E-08	2.16E-05
X6R4W8	BUB3-interacting and GLEBS motif-containing protein ZNF207 (ZNF207)	5.05E-08	1.48E-05
**Comparison between 0 Gy vs. 2 Gy at 0.023% FDR**			
A0A087WZM2	Ribonuclease T2 (RNASET2)	6.72E-07	1.02E-04
P04083	Annexin A1 (ANXA1)	2.44E-08	7.40E-06
P08133	Annexin A6 (ANXA6)	1.82E-07	3.56E-05
P12814	Alpha-actinin-1 (ACTN1)	3.05E-10	2.08E-07
P17612	cAMP-dependent protein kinase catalytic subunit alpha (PRKACA)	8.53E-11	7.77E-08
P19367	Hexokinase-1 (HK1)	3.45E-07	5.89E-05
P20073	Annexin A7 (ANXA7)	1.12E-07	2.54E-05
P21283	V-type proton ATPase subunit C 1 (ATP6V1C1)	1.74E-07	3.56E-05
P21291	Cysteine and glycine-rich protein 1 (CSRP1)	4.73E-07	7.61E-05
P22570	NADPH:adrenodoxin oxidoreductase, mitochondrial (FDXR)	3.45E-13	4.71E-10
P46777	60S ribosomal protein L5 (RPL5)	1.46E-06	2.00E-04
Q01831	DNA repair protein complementing XP-C cells (XPC)	2.19E-07	4.00E-05
Q07812	Apoptosis regulator BAX (BAX)	3.72E-08	1.02E-05
Q92466	DNA damage-binding protein 2 (DDB2)	3.60E-09	1.64E-06
Q96HC4	PDZ and LIM domain protein 5 (PDLIM5)	1.73E-08	6.77E-06
Q9H2G4	Testis-specific Y-encoded-like protein 2 (TSPYL2)	2.86E-16	7.82E-13
Q9NRX4	14 kDaphosphohistidine phosphatase (PHPT1)	2.73E-09	1.49E-06
Q9Y2W1	Thyroid hormone receptor-associated protein 3 (THRAP3)	1.09E-07	2.54E-05
Q9Y490	Talin-1 (TLN1)	2.34E-08	7.40E-06
X6R4W8	BUB3-interacting and GLEBS motif-containing protein ZNF207 (ZNF207)	1.39E-06	2.00E-04

[a]Data were analysed using one-way ANOVA test

Supplementary Table S3. Correlation between protein expression and irradiated dose

Gene symbol[a]	correlation coefficient [b]	SE[b]	*p* value[b]
**Up-regulated proteins**			
PRKACA	0.90	0.14	0.00013
ACTN1	0.89	0.12	2.25E-06
GMPR2	0.88	0.16	0.00034
FDXR	0.87	0.12	2.61E-06
CRELD2	0.84	0.18	9.60E-04
DDB2	0.78	0.13	9.72E-06
RNASET2	0.77	0.14	1.67E-05
BAX	0.76	0.14	2.31E-05
XPC	0.76	0.16	2.20E-04
TSPYL2	0.73	0.15	8.39E-05
PDLIM5	0.73	0.16	2.90E-04
GIMAP1	0.71	0.19	3.10E-03
**Down-regulated proteins**			
SF3A3	-0.81	0.13	3.55E-06
DDX21	-0.8	0.13	3.91E-06
HBB	-0.8	0.13	4.35E-06
HNRNPU	-0.78	0.13	1.21E-05
HBA1	-0.78	0.13	1.22E-05
BCLAF1	-0.77	0.14	1.50E-05
HNRNPAB	-0.75	0.14	4.06E-05
PRPF40A	-0.75	0.14	4.12E-05
RBM14	-0.75	0.14	4.16E-05

[a]Proteins with strong correlation (|correlation coefficient|>0.70, p value< 0.05) were listed.
[b]Correlation coefficient, Standard error (SE) of correlation coefficient and *p-value* was obtained by Pearson correlation analysis.

Supplementary Table S4. Protein biomarker models ranked according to AICc score

Rank[a]	Model	AICc	Weights	R^2 (*p* value)[b]	MAE[c]
1	Dose ~ FDXR+ACTN1	-13.337	0.809	0.958 (<0.0001)	0.13
2	Dose ~ FDXR+ACTN1+DDB2	-9.724	0.133	0.959 (<0.0001)	0.12
3	Dose ~ FDXR+ACTN1+BAX	-7.958	0.055	0.958 (<0.0001)	0.13
4	Dose ~ FDXR+ACTN1+DDB2+BAX	-2.306	0.003	0.942 (<0.0001)	0.15
5	Dose ~ ACTN1	11.471	0.000	0.712 (<0.0001)	0.28
6	Dose ~ FDXR	14.151	0.000	0.724 (<0.0001)	0.34
7	Dose ~ ACTN1+DDB2	14.752	0.000	0.701 (<0.0001)	0.29
8	Dose ~ ACTN1+BAX	14.833	0.000	0.713 (<0.0001)	0.28
9	Dose ~ FDXR+DDB2	14.974	0.000	0.779 (<0.0001)	0.29
10	Dose ~ FDXR+BAX	15.056	0.000	0.781 (<0.0001)	0.28
11	Dose ~ FDXR+DDB2+BAX	18.046	0.000	0.796 (<0.0001)	0.27
12	Dose ~ ACTN1+DDB2+BAX	18.632	0.000	0.707 (<0.0001)	0.28
13	Dose ~ DDB2	39.214	0.000	0.427 (0.0005)	0.45
14	Dose ~ BAX+DDB2	41.053	0.000	0.429 (0.0005)	0.45
15	Dose ~ BAX	42.756	0.000	0.391 (0.0011)	0.49

[a]Models were ranked according to AICc values.
[b]R^2 and *p* value was obtained by linear regression analysis for predicted vs. actual doses.
[c]MAE values were used as indicators to compare the difference between actual irradiated and predicted dose.

Supplementary Table S5. Dose prediction in single models using the top 4 best candidates[a]

	Predicted dose (Mean ± SD, Gy) Actual irradiated dose					
	0Gy	1Gy	2Gy	coefficient [c]	R^2 (*p* value)[b]	MAE[c]
FDXR	0.09 ± 0.19	1.15 ± 0.30	1.63 ± 0.57	0.77 ± 0.12	0.72 (<0.0001)	0.34
BAX	0.27 ± 0.23	1.16 ± 0.69	1.24 ± 0.31	0.49 ± 0.13	0.39 (0.0011)	0.49
DDB2	0.23 ± 0.25	1.16 ± 0.73	1.31 ± 0.30	0.55 ± 0.14	0.43 (0.0003)	0.45
ACTN1	0.08 ± 0.13	1.16 ± 0.55	1.58 ± 0.25	0.76 ± 0.12	0.71(<0.0001)	0.28

[a]Data obtained from proteomic analysis were used for investigating dose prediction.
[b]Coefficient, R^2 and *p* value was obtained by linear regression analysis for predicted vs. actual doses.
[c]MAE values were used as indicators to compare the difference between actual irradiated and predicted dose.
